# Statistical Study of the Method of Radius-Change Sphericity Measurements

**DOI:** 10.3390/s21134563

**Published:** 2021-07-03

**Authors:** Krzysztof Stępień, Dariusz Janecki, Stanisław Adamczak

**Affiliations:** 1 Department of Manufacturing Engineering and Metrology, Kielce University of Technology, Al. 1000-lecia P. P. 7, 25-314 Kielce, Poland; adamczak@tu.kielce.pl; 2 Centre for Laser Technologies of Metals, Kielce University of Technology, Al. 1000-lecia P. P. 7, 25-314 Kielce, Poland; djanecki@tu.kielce.pl

**Keywords:** spherical part, measurement, radius-change method, form deviation, metrology of geometrical quantities

## Abstract

At the Kielce University of Technology, a concept of the accurate measurement of sphericity deviations of machine parts has been developed. The concept is based upon the measurement of roundness profiles in many clearly defined cross-sections of the workpiece. Measurements are performed with the use of a typical radius change measuring instrument equipped with a device for accurate positioning of the ball. This paper focuses on the statistical analysis of the differences between measurement results of spherical parts obtained by the new and traditional method. The differences were analyzed by applying preselected statistical parameters and diagrams. Results of the analysis revealed that that the new method may contribute to a much more reliable measurement of form deviations of spherical parts.

## 1. Introduction

Spherical elements constitute a significant group of machine parts. In particular, they are common in the bearing industry, where they have to meet high quality requirements. One of the most important factors regarding the quality of spherical parts are form deviations. 

In contemporary industrial practice, the form deviations of spherical parts are determined through the measurement of the roundness deviation in a random cross-section of the sphere. The dominant method used in the industry to measure roundness deviation is the so-called radius-change method, in which a sensor detects variations in the radius of the measured part [1]. 

It is obvious that such a measurement technique does not enable obtaining reliable information about the whole investigated surface, because a large fragment of the surface is not sampled.

Therefore, the authors undertook research aimed at developing a new method of measuring spherical elements, which would enable a more reliable assessment of form deviations of such parts. These studies were conducted in close cooperation with the Rolling Bearings Factory in Kraśnik (Poland). The research activities resulted in the development of a system that could measure the sphericity deviation of bearing balls using a highly accurate radius-change system. The authors’ aim was to develop a method to be practically useful in industry. Therefore, a comparative study of the new method with the traditional method were carried out under conditions closest to real industrial conditions. The aim of this research was to determine to what extent the results obtained with the new method differed from the results obtained with the traditional method, and an additional goal was to investigate the nature of these differences. In Section 2 of this paper, the state of the art of the measurement and evaluation of form deviations of spherical elements is presented. Section 3 describes the developed measurement concept. Section 4 presents the experiment. Section 5 and Section 6 describe the results of the experiments and their statistical analysis. The last section of this paper contains conclusions and directions for further research.

## 2. State of the Art of Sphericity Measurements

As mentioned in the previous section, an evaluation of form deviations of spherical parts in industry is usually performed by so-called radius-change measuring instruments equipped with a contact sensor [1]. 

Apart from radius change methods with a contact sensor, intensive research has been conducted in the field of optical measurements. It is noteworthy that the first automated system dedicated to the optical measurement of spherical parts was produced in Czechoslovakia in the 1960s for the bearing industry. The operation of the system was based on an analysis of the changes in the intensity of the light beam reflected from the spherical surface [2].

Currently, methods based on using laser interferometers for accurate measurements of the surface topography of spherical parts are intensively studied, which are described elsewhere [3]. 

The authors of the abovementioned study present a system that measures small regions of a sphere with the use of a Fizeau interferometer. The measured regions are then numerically joined (this operation is called *stitching*) together to assess the topography of the whole surface of the sphere. In this method, it is essential to accurately determine the position of the sphere. Therefore, the fundamental parts of the system are the units that control the change of the position of the sphere during measurement. 

A similar system is described in [4]. This system uses an appropriately modified Fizeau interferometer, and small fragments of the sphere are measured in the subsequent stages of the measurement. The method described in [4] requires, similarly to the method proposed in [3], numerical stitching of small fragments of the sphere, and the topography of the sphere is reconstructed from small fragments. 

The authors of [5] dealt with the form deviation measurements of the spherical part of electrodes used in the electro-discharge machining process. The measurements were performed with the use of an appropriately calibrated camera that captures images of the spherical tip of the electrode. The system saves the image of the electrode, and the relevant software computes the coordinates of the points lying on the edge of the image of the electrode. Then, the electrode is rotated to the next position and the procedure of computing coordinates of the points lying on the edge of the image is repeated. This way, a matrix containing coordinates of the points of the tip of the electrode is obtained. On the basis of these coordinates, the form deviations of the spherical part of the electrode are then determined. 

Interesting solutions of the problem of sphericity measurements by optical method have been presented. The authors of [6] proposed the application of digital image processing methods to obtain a 3D view of a surface which is reconstructed from 2D images. It is obvious that, on the basis of a singular 2D image, only a half-sphere can be reconstructed. In this method, firstly, a 2D image of the surface is captured with the use of a pre-calibrated camera. The distances of individual points of the surface from the camera correspond to changes in the intensity of the grey scale of the captured image. The analysis of the changes in the intensity contributes to the generation of a 3D view of the studied surfaces. 

It is noteworthy that various research centers and universities have investigated the problem of applying coordinate measuring machines to accurately measure form deviations of spherical parts. Taking into account the dynamic development of coordinate metrology, it can be assumed that, in the near future, measurements of spheres with the use of CMMs will be one of the leading methods in industry. However, currently, coordinate measuring machines do not offer measurement accuracy that fulfils all the requirements of manufacturers of very precise machine parts (for example, manufacturers of rolling bearings, eyeglasses, etc.). 

Apart from investigating the methods of measuring form deviations of spherical parts, research activities aiming to develop new methods of sphericity evaluation have also been carried out. Usually, in the evaluation of sphericity deviations, the methodology is used that is analogical to that applied in the case of evaluating roundness deviation. The main difference is that for the evaluation of roundness deviation, 2D features and parameters are used, whereas for the evaluation of sphericity deviation, analogical 3D features and parameters are applied. For example, minimum zone circles for roundness evaluations correspond to minimum zone spheres for the sphericity evaluation.

Studies of the evaluation of sphericity deviations include, for example, computing various types of reference features from measurement data. Various methods can be applied for this purpose, for example, the numerical processing of coordinate measurement data [7,8,9]. 

Another way to calculate parameters of reference features are computational geometric techniques. Usually, such techniques use so-called Voronoi diagrams, which have been described in detail elsewhere [10,11]. 

Other methods of evaluating sphericity deviations that are noteworthy are a method applying the theory of minimum potential energy [12] and a method based on the analysis of statistical parameters [13].

It is worth noting that spherical specimens are also used in the so-called random ball test. The random ball test (RBT) is a technique for calibrating spherical wavefronts. In this test, a high-quality ball is measured against the reference surface at its confocal position. Measurements of the sphere are conducted in a number of random orientations. Such an approach averages out errors due to the surface irregularity of the ball [14,15].

However, it should be noted that spherical parts used in bearing industry have much larger surface irregularities than balls used in RBT.

## 3. Concept of Radius Change Measurement of Sphericity 

**Figure 1:**
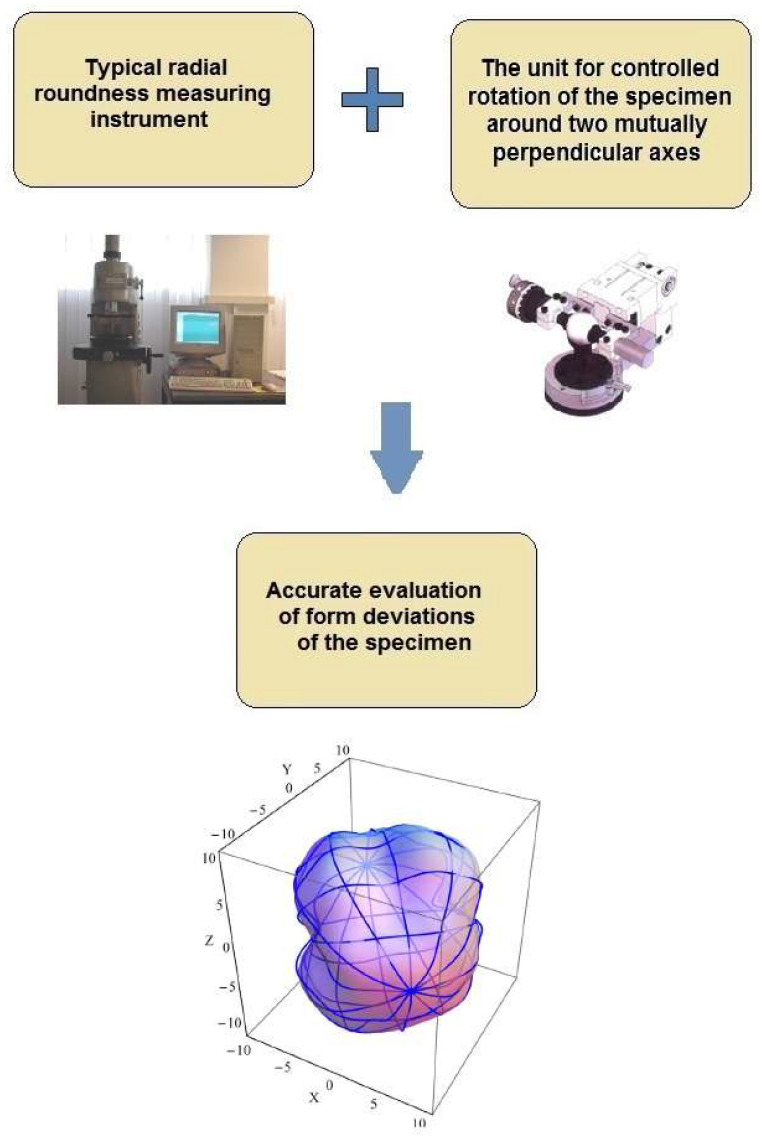
The concept of the sphericity measurement by the radius-change instrument.

The concept of using a typical radius-change instrument to measure sphericity deviations of machine parts was as follows: A typical radius-change measuring instruments commonly applied in the bearing industry, for example, Talyrond 73, was used. Additionally, we equipped the measuring instrument with the unit allowing precise controlled rotation of the sphere. With this unit, we could perform roundness measurements in clearly defined cross-sections of the measured parts. After a series of measurements, we sent the measurement data to the software that combined the measured 2D profiles into a 3D model of the part. The concept is presented in Figure 1. 

The concept requires solving a number of various computational problems, such as profile matching and filtering or computing of the reference sphere. 

The main reason for computational difficulties is that the sphericity deviation is a 3D problem. The area of measurement and evaluation of roundness deviations, which is a 2D problem, is quite well recognized. Roundness deviations can be conveniently written using polar coordinates *R* and *φ*, where *R* is the radius at a given point of the roundness profile, the position of which is described by the angular coordinate *φ*.

Assuming that the radius of a measured circle is much larger than coordinates of the origin of the circle *e_x_* and *e_y_*, one can write the equation of the reference circle as follows:

(1) $$R_{ref}\left(\varphi \right)=R_{0}+e_{x}\cos\varphi +e_{y}\sin\varphi$$

where *R*_0_ is the mean value of *R*(*φ*), and *e_x_*, *e_y_*, and *e_z_* are the coordinates of the origin of the circle.

Considering Equation (1), a distance of any given point of the roundness profile *R* (*φ*) from the reference circle can be written as follows:

(2) $$\Delta R\left(\varphi \right)=R\left(\varphi \right)-R_{ref}\left(\varphi \right)$$

Solving the problem of measuring sphericity deviations requires a transition from 2D to 3D. Therefore, it is most convenient to analyze this problem using the spherical coordinates *S*, *φ*, *θ*, where *S* is the radius of the sphere at a given point whose position is described by the two angular coordinates *φ* and *θ*.

Assuming that the radius of the measured sphere is much larger than coordinates of the origin of the sphere *e_x_*, *e_y_*, *e_z_*, the equation of the reference sphere can be written as follows:

(3) $$S_{ref}\left(\theta ,\phi \right)=S_{0}+e_{x}\cos\varphi \sin\theta +e_{y}\sin\varphi \cos\theta +e_{z}\cos\theta$$

where *S*_0_ is the mean value of *S*, and *e_x_*, *e_y_*, and *e_z_* are the coordinates of the origin of the sphere.

Considering Equation (3), a distance of any given point of the spherical surface *S*(*φ*,*θ*) from the reference sphere can be written as follows:

(4) $$\Delta S\left(\theta ,\varphi \right)=S\left(\theta ,\varphi \right)-S_{ref}\left(\theta ,\varphi \right)$$

**Figure 2:**
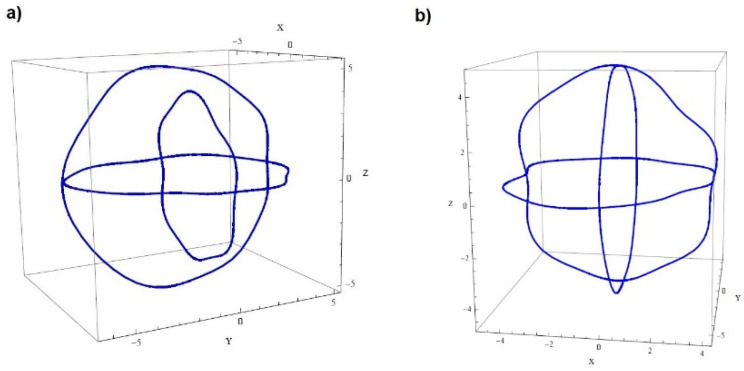
Significance of the profile matching: (**a**) raw measurement data (unmatched profiles); (**b**) profiles after matching.

It should be emphasized that the method presented by the authors makes it impossible to easily generalize the equations related to the deviation of roundness into a 3D space. The primary reason for this is that the radius-change measurement is a relative assessment. The measuring instrument does not record the coordinates of the measurement points in the global system; therefore, the roundness profiles in various cross sections of the same part may have significantly different mean values. If we plot the raw measurement data on the diagram, we obtain distorted results, as shown in Figure 2a. 

Therefore, it is necessary to perform so-called profile matching. To match the profiles, the coordinates of the intersection of the profiles should be determined first. We know that the values of matched profiles in the points of the intersections should be equal. Using this fact and appropriate functions (e.g., the LinearSolve function of Wolfram Mathematica), we can correct the values of the profiles so that they match each other, as shown in Figure 2b. 

The solution of the problem of profiles matching is explained in detail in [16].

A full mathematical model of the method has been presented in [17], whereas an example of its practical application for singular spherical part has been published in [18]. In addition, practical verification of the method under industrial conditions has been given in [1].

The practical part of the work included the design and construction of the unit for the controlled positioning of the measured sphere. Successful solving of the abovementioned theoretical and practical problems made it possible to evaluate the deviations of the spherical part on the basis of the set of roundness profiles measured in clearly defined cross-sections. There were various measuring strategies tested during the experiment, and finally, the “cage” strategy was selected to be applied for verification of the concept under industrial conditions. 

## 4. Experiment

To verify a concept experimentally, a system for the accurate positioning of spherical parts was designed and constructed. The system has been described in detail in [18]. 

**Figure 3:**
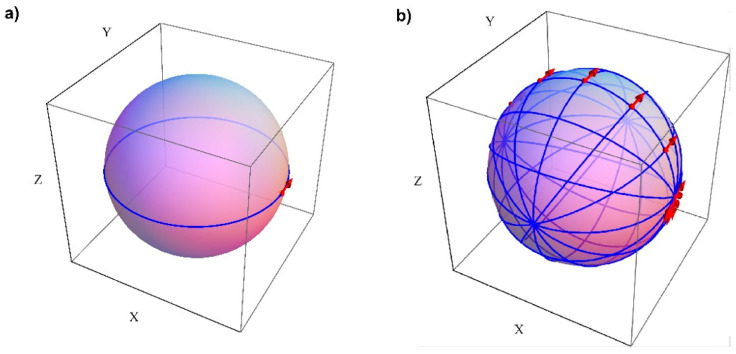
Measurement strategies used in the experiment: (**a**) the traditional strategy; (**b**) the new strategy.

The fundamental part of the system is the unit for the controlled rotation of spherical parts. The unit was designed to be mounted on a measuring table of a Talyrond 73. The experiment had two stages: in each stage, a set of 23 spherical parts was measured by the traditional and by the new method, and the aim of the measurements was to determine form deviations of the parts. The traditional method was that commonly applied in industry, i.e., roundness deviation in a random cross-section of the sphere was measured. The diameter of the measured spheres was equal to 50.8 mm. Using the new method enabled the observation of sphericity deviations of the parts. Figure 3 shows the traditional and the new measurement strategies. 

After each measurement, data were saved in the computer memory and then transferred to the software, enabling the accurate analysis of sphericity deviations. All procedures allowing the processing of measurement data were developed in Mathematica. Source codes of the selected functions are given in the Appendix A.

Measurements by the traditional method were performed with the use of the instrument MWA series C, by SKF. There were 1024 sampling points taken in one cross-section. The rotational speed of the sensor was equal to 9.837 rev./min. The reference was the least squares circle. The value of the roundness deviation was determined for the unfiltered profiles. For each part, a diagram of the measured profile was plotted as well as a bar chart of the harmonic components. The maximum number of harmonic components was 128.

## 5. Results

For the traditional method, the total roundness deviation RONt was determined, whereas for the new method, the total sphericity deviation was determined. The results of the measurements are given in Table 1 and Table 2.

**Figure 4:**
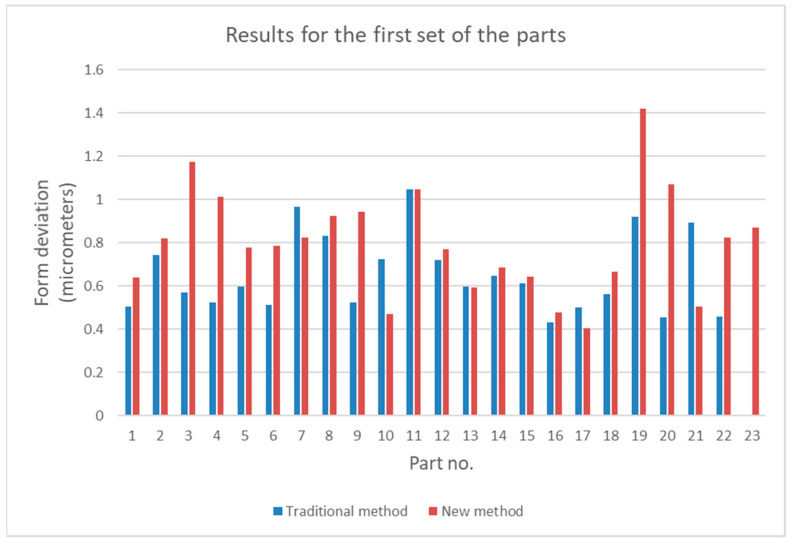
Comparison of form deviations obtained by the traditional and by the new method for the first set of parts.

**Figure 5:**
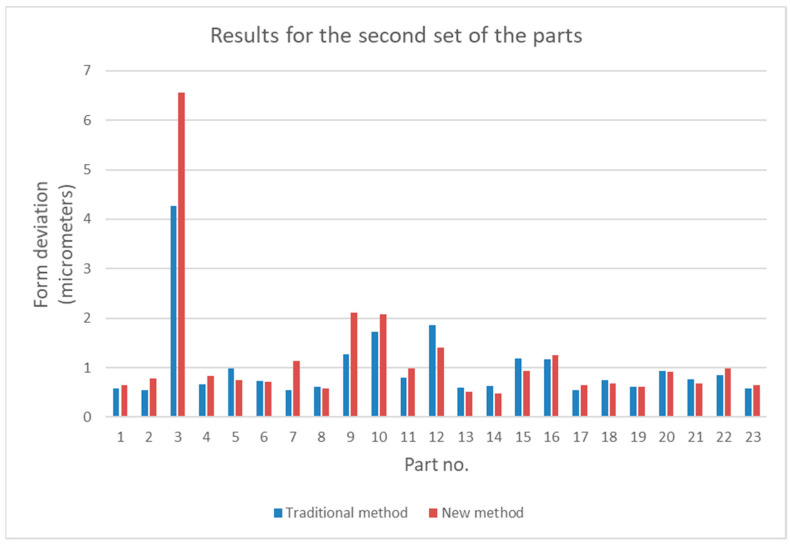
Comparison of form deviations obtained by the traditional and by the new method for the second set of parts.

The results given in Table 1 and Table 2 are presented graphically in Figure 4 and Figure 5.

Diagrams shown in Figure 4 and Figure 5 show that values of form deviations obtained by the traditional and the new method can differ significantly. To investigate this difference more precisely, statistical analysis was conducted; this is presented in the next section.

## 6. The Statistical Analysis of the Results and Discussion

The statistical analysis of the results was divided into two parts. The first part concerned the comparison of the results obtained with the traditional method and the new method. The second part focused on the study of the differences between the results of roundness measurements in various cross-sections of the same elements.

### 6.1. The Analysis of Differences between the Results Obtained by the New and Traditional Methods

The measurement results given in Table 1 and Table 2 were investigated with the use of statistical parameters and tools [19]. For the measurement results given in Table 1 and Table 2, the relative difference *diff_r_* between the results was calculated according to Equation (5):

(5) $$diff_{r}=\left|\frac{D_{t}-D_{n}}{D_{t}}\right|\cdot 100\%$$

where *Dt* is the form deviation obtained by the traditional method, and *Dn* is the form deviation obtained by the new method.

**Figure 6:**
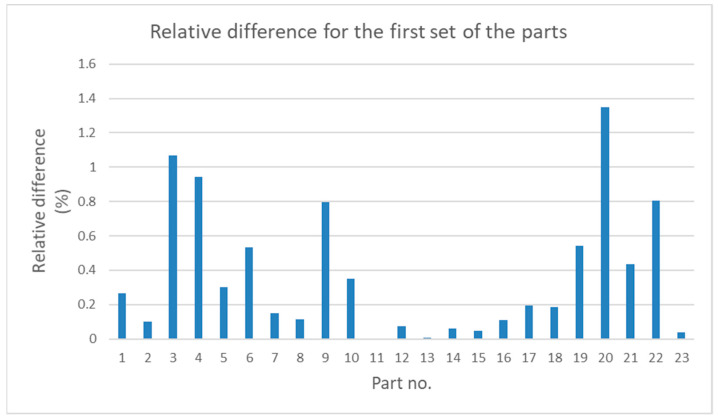
Relative differences between the values of deviations obtained with the use of the new and traditional methods for the first set of parts.

**Figure 7:**
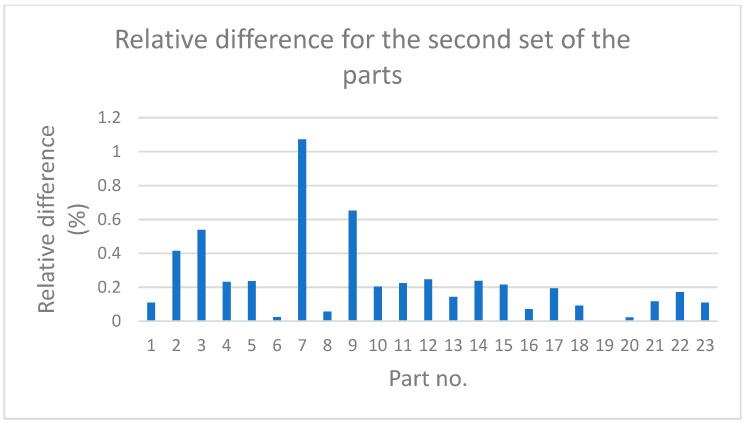
Relative difference between the values of deviations obtained with the use of the new and traditional methods for the second set of parts.

Figure 6 and Figure 7 show the values of relative differences calculated according to Equation (5) for both sets of parts. 

Then, the statistical parameters related to the values of the relative differences between the results for both sets of parts were calculated. In this study, the following statistical parameters were analyzed: mean value, maximum value, minimum value and the standard deviation. The values of the parameters mentioned above are given in Table 3.

The next stage of the study was to conduct a statistical comparison of mean values for the results of both measurement series. To conduct this test, the following formula was used:

(6) $$t=\frac{x_{av1}-x_{av2}}{\sqrt{\frac{s_{1}{}^{2}+s_{2}{}^{2}}{n}}}$$

where *x_av_*_1_ is the mean value of relative difference for the first set of parts, *x_av_*_2_ is the mean value of relative difference for the second set of parts, *s*_1_ is the standard deviation of the relative differences for the first set of parts, *s*_2_ is the standard deviation of the relative differences for the second set of parts, and *n* is the number of parts in one set (n=23).

After inserting the relevant values into Equation (6), we obtained:

(7) $$t=\frac{36.8-25.4}{\sqrt{\frac{38.2^{2}+25^{2}}{23}}}≅1.20$$

For the test, a confidence level α=0.05 was assumed and the critical value was $t_{\alpha }=1.717$. 

As shown, the experimental value of *t* calculated with the use of Equation (6) (t=1.717) did not exceed the critical value $t_{\alpha }=1.717$. Thus, the test indicates that the difference between mean values of the relative difference for both sets of the parts is not statistically significant.

**Figure 8:**
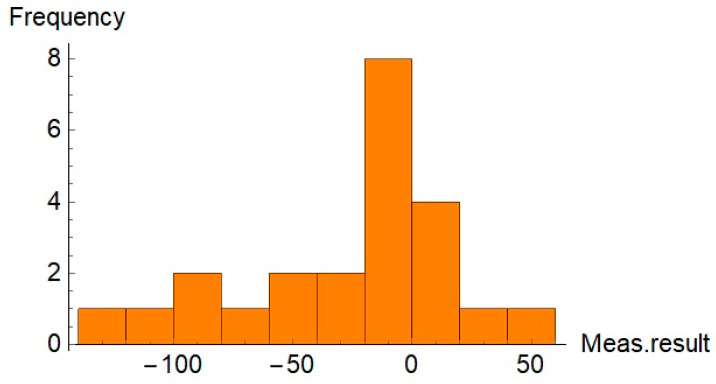
Histogram of the relative differences between compared results for the first set of the parts.

**Figure 9:**
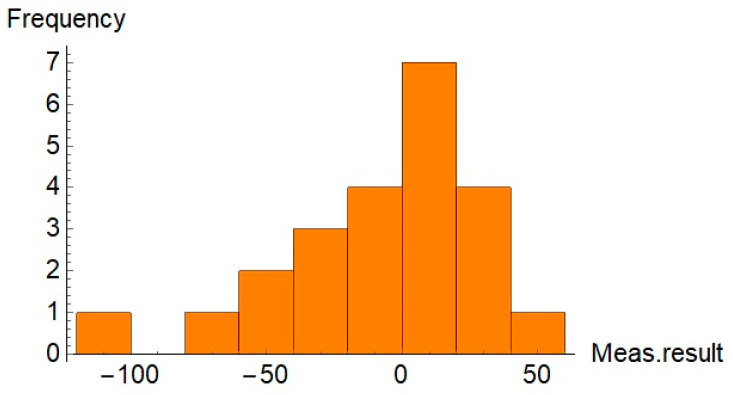
Histogram of the relative difference between compared results for the second set of the parts.

To evaluate the distribution of the relative differences between results for both sets of the parts, histograms were generated. The histograms are shown in Figure 8 and Figure 9. 

Additionally, the function FindDistribution of Wolfram Mathematica was used to find a statistical distribution that fitted the data shown in Figure 8 and Figure 9. Results of the application of the function FindDistribution to measurement data are given in Table 4.

Information about the distribution of results can be used to evaluate the confidence intervals of the results for both sets of data. 

For the first set of data, the confidence interval Δ_1_ is given by Equation (8):

(8) $$\Delta_{1}=\pm \frac{a}{2\sqrt{3}}$$

where *a* is the distribution range. For the measurement data of the first set, the distribution range was equal to a=178.47%.

For the second set of data, the confidence interval Δ_2_ is given by Equation (9):

(9) $$\Delta_{2}=\pm k\cdot s$$

where *k* is the coverage factor attributed to the assumed distribution and the probability level. For normal distribution and for probability level P=0.95, the value of the coverage factor k=1.96. *s* is the standard deviation in a given set. For the measurement data of the second set, the standard deviation was equal to s=25%.

Table 5 provides the values of the confidence intervals for the measurement results of both sets of parts. 

Values given in Table 5 show that the confidence intervals for both measurements of both sets of parts are similar despite the fact that the assumed distributions are different.

Measurement data presented in Table 1 and Table 2 and in Figure 4 and Figure 5 show that, for some parts, in both sets, the differences between the measurement results obtained by the traditional way and by the new method are significant. It is noticeable that in most cases the relative difference did not exceed 50%; however, for some parts they were greater than 100%. Analysis of the values given in Table 3 shows that for the first set, the mean value, the maximum value, and the standard deviation were about 50% higher than the relevant values for the second set. The minimum values of *diff_r_* for both sets of data were very similar. Despite the differences of basic statistical parameters, the result of the test of mean values indicated that the difference between the mean values of *diff_r_* was not statistically significant. Results of the operation of the function FindDistribution (Wolfram Mathematica package) indicated that best fitting distributions for both sets of data were as follows: uniform distribution for the first set and normal distribution for the second set. Nevertheless, the confidence intervals evaluated for both sets were quite similar (51%, 56% for the first set, and 49% for the second set).

### 6.2. Analysis of Differences in Values of the Roundness Deviation in Various Cross-Sections of the Same Spherical Part

**Figure 10:**
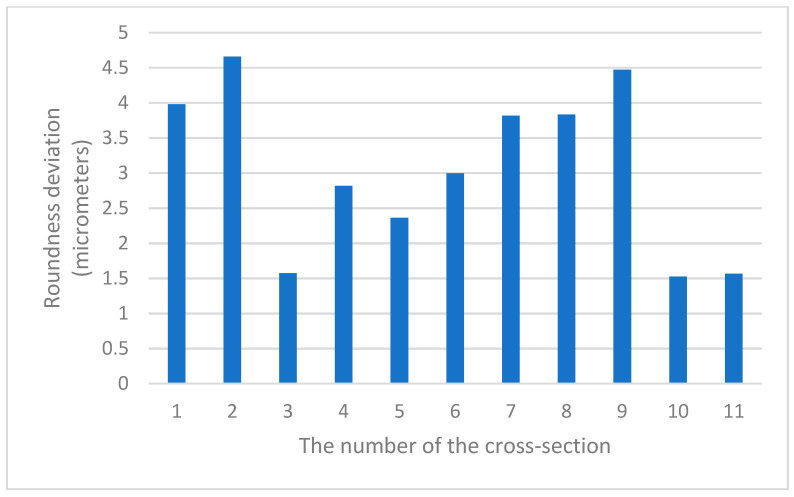
Values of roundness deviations in subsequent cross-sections of sphere no. 3 from the first dataset.

The presented method of sphericity measurements is based on the numerical joining of roundness profiles measured in a number of strictly defined cross-sections of a sphere (we call this operation *profile matching*). Such an approach made it possible to conduct a comparative analysis of the values of the roundness deviation in various cross-sections of the same part. Figure 10 shows the values of the roundness deviation in various cross-sections of one of the measured parts (it was the third part in the first dataset).

The diagram shown in Figure 10 shows that the values of roundness deviation obtained in various cross-sections of the measured part differed significantly. Therefore, statistical evaluations of the differences in the roundness deviation values in individual cross-sections for each of the measured spheres were performed. For this purpose, the following parameters were determined for each part: minimum value, maximum value, range, mean value and standard deviation. The results of the calculated parameters are presented in Table 6 and Table 7.

**Figure 11:**
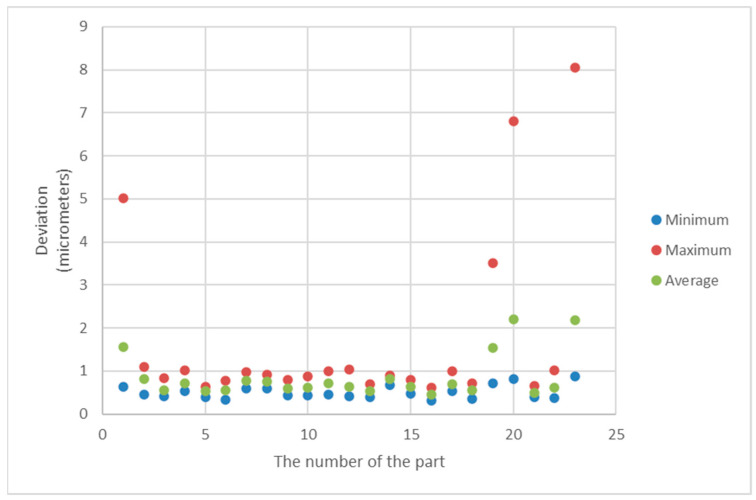
Minimum, maximum and average values of the form deviation for each sphere from the first dataset.

**Figure 12:**
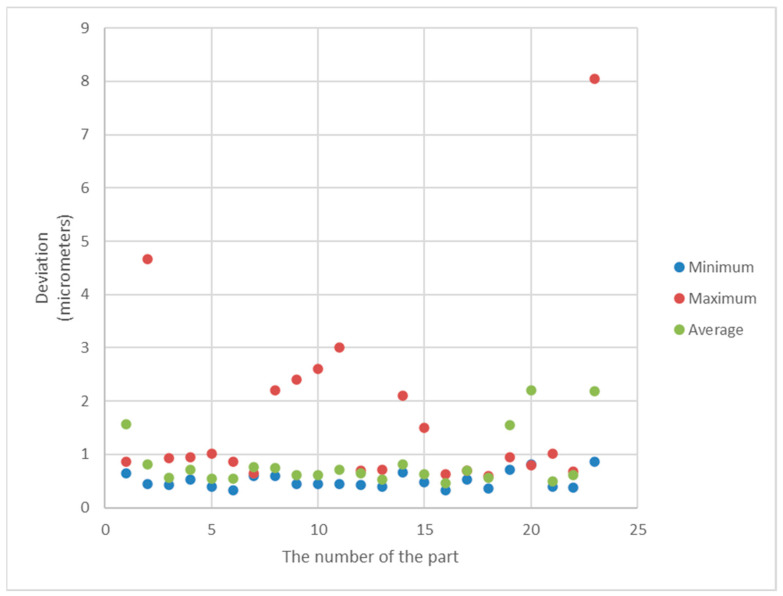
Minimum, maximum and average values of the form deviation for each sphere from the second dataset.

Figure 11 and Figure 12 show the minimum, maximum and average values of the roundness deviation for each sphere from the first and second datasets, respectively.

**Figure 13:**
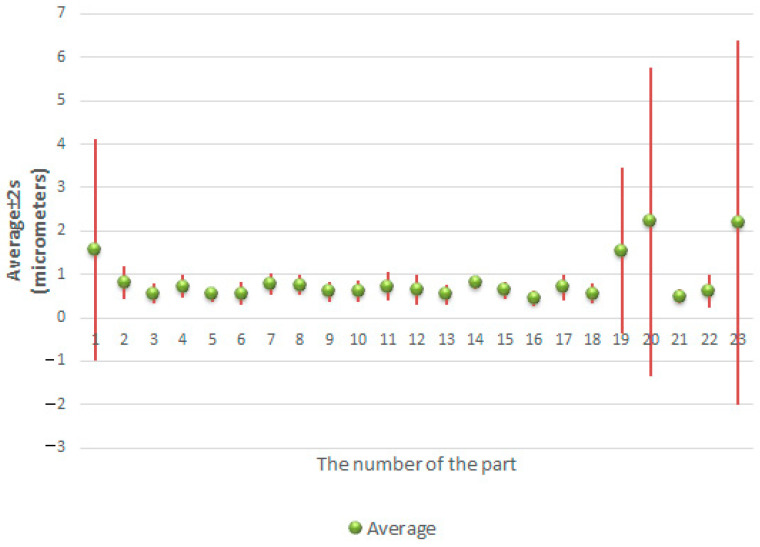
Average values of the roundness deviation and their confidence levels (calculated as ± 2 *s*) for each sphere from the first dataset.

**Figure 14:**
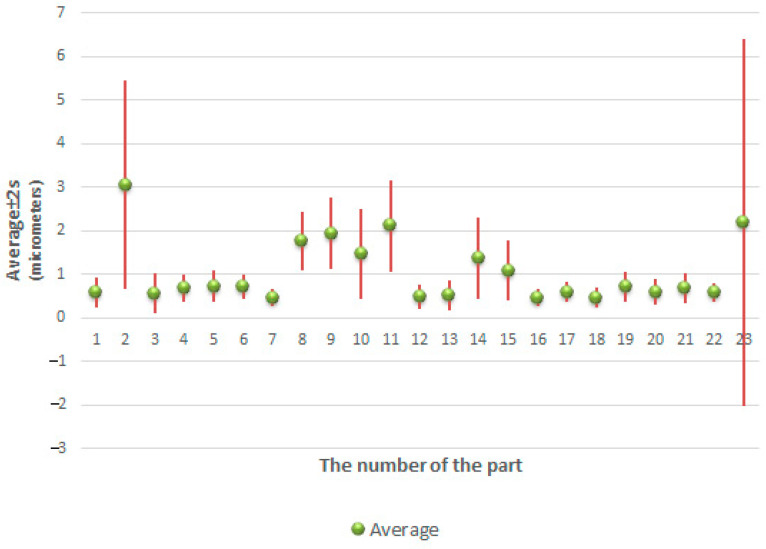
Average values of the roundness deviation and their confidence levels (calculated as ± 2 *s*) for each sphere from the second dataset.

Accordingly, Figure 13 and Figure 14 show the average values of the deviations along with their confidence intervals calculated as ±2 *s*, where *s* is the standard deviation.

The results presented in Table 6 and Table 7 and the graphs presented in Figure 11, Figure 12, Figure 13 and Figure 14 clearly show that there are very large differences in the values of roundness deviation in various sections of the same sphere. 

It proves that the value of the roundness deviation for a given part may be very dependent on how the sphere is oriented initially. In the method of sphericity measurements proposed by the authors, the roundness profiles in many cross-sections of the sphere are analyzed and numerically joined. Therefore, in our opinion, this method is a more consistent way of evaluating of form deviations of spherical parts during the manufacturing process.

## 7. Conclusions

In previously published studies [16,17,18], researchers have presented the concept of measurements of form deviations of spherical parts with the use of the radius-change method. For the new method, a much larger area of the part is covered with sampling points than for the traditional method. In addition, the radius-change measuring instruments are highly accurate. Therefore, it is reasonable to assume that the new method gives more reliable results than the traditional technique. The aim of the study presented in this paper was to quantitatively assess to what extent the measurement with the new method is more accurate than the measurement with the traditional method. The analysis was performed with the use of statistical tools and parameters for the two sets of data. It is noticeable that for both sets, the results obtained by the traditional and by the new method differed significantly. In addition, the conducted research has shown that the results of measurements made using the traditional method may be strongly dependent on the initial position of the ball.

Thus, the new method can be recommended as a process that provides more reliable results of measurements of spherical parts. 

Results of the experiments show that the proposed method is quite easy to apply. Additionally, it can be more user-friendly if the process of rotation of the spherical elements is automated. Considering the possibilities of practical application of the described method, one should note that the proposed measurement system is relatively simple. It requires accurate instruments for radial roundness measurements and a unit for the controlled rotation of measured spheres. Radial roundness measurement instruments are widespread; therefore, the concept could be easily applied in industrial situations.

In further research on the new method, the authors will focus on determining the uncertainty of the measuring systems. This will be performed using a spherical standard. First, the authors will deal with the calculation of type A uncertainty. Next, the plan is to focus on type B uncertainty.

## Figures and Tables

**Table 1:** Measurement results for the first set of parts.

Form Deviation Measured by the Traditional Method (Micrometers)	Form Deviation Measured by the New Method (Micrometers)
0.505	0.64
0.743	0.819
0.568	1.175
0.522	1.014
0.597	0.778
0.513	0.786
0.968	0.824
0.83	0.923
0.525	0.943
0.723	0.471
1.046	1.048
0.719	0.771
0.597	0.592
0.647	0.685
0.613	0.641
0.432	0.479
0.499	0.403
0.561	0.666
0.922	1.42
0.456	1.072
0.892	0.505
0.457	0.825
0.903	0.87

**Table 2:** Measurement results for the second set of parts.

Form Deviation Measured by the Traditional Method (Micrometers)	Form Deviation Measured by the New Method (Micrometers)
0.585	0.649
0.548	0.775
4.267	6.566
0.669	0.824
0.975	0.745
0.725	0.708
0.545	1.129
0.615	0.581
1.275	2.107
1.731	2.084
0.799	0.978
1.855	1.399
0.598	0.512
0.628	0.479
1.193	0.936
1.175	1.259
0.772	0.329
0.545	0.65
0.746	0.678
0.607	0.606
0.936	0.916
0.772	0.682
0.843	0.987

**Table 3:** Statistical parameters of the relative difference between results obtained by new and traditional methods for the first set of parts.

Parameter Name	Parameter Value for the first Set of the Parts (%)	Parameter Value for the second Set of the Parts (%)
**Mean value of the relative difference**	36.8	25.4
**Maximum value of the relative difference**	135.1	107.2
**Minimum value of the relative difference**	0.19	0.16
**Standard deviation**	38.2	25

**Table 4:** Results of the application of the function FindDistribution to measurement datasets.

The Number of the Measurement Dataset	Distribution Type and Its Parameters
**First set**	Uniform distribution (min. value: −135.088, max. value: 43.3857)
**Second set**	NormalDistribution [mean value: −9.66936, standard deviation: 40.513]

**Table 5:** Confidence intervals for measurement results of both sets of parts.

The Number of the Measurement Dataset	Confidence Interval
First set	$\Delta_{1}=\pm \frac{178.47}{2\sqrt{3}}\%=51.58\%$
Second set	$\Delta_{2}=\pm 1.96\cdot 25\%=49\%$

**Table 6:** Statistical parameters describing the differences between form deviations in subsequent cross-sections of each sphere from the first dataset (all values are given in micrometers).

Minimum Value	Maximum Value	Range	Average Value	Standard Deviation
0.641	5.01	4.369	1.561	1.275
0.451	1.105	0.654	0.814	0.186
0.42	0.841	0.421	0.556	0.118
0.531	1.021	0.49	0.717	0.134
0.401	0.634	0.233	0.539	0.082
0.323	0.781	0.458	0.554	0.130
0.593	0.986	0.393	0.768	0.125
0.601	0.911	0.31	0.752	0.113
0.437	0.805	0.368	0.604	0.114
0.442	0.882	0.44	0.610	0.129
0.452	0.991	0.539	0.716	0.163
0.421	1.032	0.611	0.643	0.172
0.391	0.693	0.302	0.534	0.112
0.671	0.896	0.225	0.806	0.074
0.471	0.79	0.319	0.630	0.096
0.321	0.617	0.296	0.460	0.091
0.526	0.993	0.467	0.694	0.148
0.362	0.71	0.348	0.561	0.113
0.705	3.504	2.799	1.549	0.958
0.811	6.801	5.99	2.207	1.785
0.401	0.658	0.257	0.490	0.079
0.381	1.012	0.631	0.605	0.184
0.87	8.055	7.185	2.186	2.095

**Table 7:** Statistical parameters describing the differences between form deviations in subsequent cross-sections of each sphere from the second dataset (all values are given in micrometers).

Minimum Value	Maximum Value	Range	Average Value	Standard Deviation
0.31	0.86	0.55	0.581	0.154
1.527	4.658	3.131	3.054	1.179
0.277	0.924	0.647	0.563	0.221
0.511	0.941	0.43	0.670	0.143
0.453	1.01	0.557	0.724	0.169
0.502	0.871	0.369	0.725	0.124
0.352	0.654	0.302	0.464	0.083
1.3	2.202	0.902	1.759	0.322
1.23	2.402	1.172	1.937	0.397
0.824	2.602	1.778	1.451	0.501
1.115	3.001	1.886	2.113	0.507
0.311	0.7	0.389	0.489	0.126
0.315	0.708	0.393	0.508	0.158
0.746	2.108	1.362	1.374	0.448
0.554	1.504	0.95	1.085	0.327
0.343	0.621	0.278	0.456	0.084
0.411	0.7	0.289	0.596	0.103
0.352	0.6	0.248	0.464	0.096
0.441	0.953	0.512	0.703	0.154
0.332	0.801	0.469	0.596	0.129
0.51	1.015	0.505	0.672	0.157
0.401	0.675	0.274	0.566	0.091
0.31	0.86	0.55	0.581	0.154

## Data Availability

Data supporting the findings of this study are available from the corresponding author on request.

## Appendix A

  Source codes of the selected functions and procedures used in the study:

  (a) Defining the “cage” measurement strategy

CombineMethod[\[Delta]_, R_List, \[Beta]1_, \[Beta]2_] :=

  Module[{\[ScriptCapitalR], Ra, Rb, i, m},

  \[ScriptCapitalR] = IdentityMatrix[3 ];

  Ra = Rz[\[Beta]1].Ry[\[Delta]].Rz[-\[Beta]1];

  Rb = Rz[\[Beta]2].Ry[\[Delta]].Rz[-\[Beta]2];

  m = {{0, 0, 0}};

  For[i = 1, i <= Length[R], i++,

  If[R[[i]] == \[ScriptA], \[ScriptCapitalR] =

  Ra.\[ScriptCapitalR], \[ScriptCapitalR] = Rb.\[

ScriptCapitalR]];

  m = m~Join~{RotMatrix2Angles[\[ScriptCapitalR]]};

  ]; m

  ]

  (b) Calculating the profile deviation

GetProfileDev[rr_, m_, \[Kappa]_] :=

  Module[{M, ms, \[CapitalPsi], \[Eta]t,

  G, \[ScriptCapitalG], \[ScriptG], \[ScriptP]ref, \

  \[CapitalDelta]rr},

  M = Length[m]; ms = Map[sub, m];

  \[CapitalPsi] = {1, Cos\[CurlyPhi]s Sin\[Theta]s,

  Sin\[CurlyPhi]s Sin\[Theta]s, Cos\[Theta]s};

  \[Eta]t = InvertedProfileDensity[m, \[Kappa]];

  G = Outer[Times, \[CapitalPsi], \[CapitalPsi]];

  \[ScriptCapitalG] =

  Sum[NIntegrate[

  G \[Eta]t[[i]] /. ms[[i]] , {\[CurlyPhi]t, 0, 2 \[Pi]}], {i, 1,

  M}];

  \[ScriptG] =

  Sum[NIntegrate[(rr[[i]] \[Eta]t[[i]] \[CapitalPsi] /.

  ms[[i]]), {\[CurlyPhi]t, 0, 2 \[Pi]}], {i, 1, M}];

  \[ScriptP]ref = Inverse[\[ScriptCapitalG]].\[ScriptG];

  \[CapitalDelta]rr =

  Table[rr[[i]] - \[ScriptP]ref.\[CapitalPsi] /. ms[[i]], {i, 1, M}];

  \[CapitalDelta]rr

  ];

  (c) Profile filtering

      ProfileFiltring[rm_, m_, \[Kappa]_, K_] :=

      Module[{ms, M, \[Eta]t, \[CapitalPhi], K1, F0, F, g,

      i, \[CapitalTheta], \[CapitalOmega], \[CapitalPsi], W1, W2,

      W, \[Rho], H, \[CapitalDelta]rf},

      ms = Map[sub, m];

      M = Length[m];

      \[Eta]t = InvertedProfileDensity[m, \[Kappa]];

      \[

     CapitalPhi] = {1};

      For[k = 1, k <= K,

      k++, \[CapitalPhi] = \[CapitalPhi]~

      Join~{Cos[k \[CurlyPhi]t], Sin[k \[CurlyPhi]t]}];

      \[CapitalPsi] = {1, Cos\[CurlyPhi]s Sin\[Theta]s,

      Sin\[CurlyPhi]s Sin\[Theta]s, Cos\[Theta]s};

      \[CapitalTheta] = {Cos[\[CurlyPhi]t], Sin[\[CurlyPhi]t], 1};

      \[CapitalOmega] = \[CapitalTheta]~Join~(-\[

     CapitalPhi]);

      K1 = 3 + 2 K + 1;

      F0 = Table[0, {K1}, {K1}];

      For[i = 1, i <= K1, i++, F0[[i, i]] = \[Pi]];

      F0[[3, 3]] = F0[[4, 4]] = 2 \[Pi];

      F0[[3, 4]] = F0[[4, 3]] = -2 \[Pi];

      F0[[5, 1]] = F0[[1, 5]] = F0[[6, 2]] = F0[[2, 6]] = -\[Pi];

      F = Table[0, {M K1}, {M K1}];

      g = Table[0, {M K1}];

      For[i = 1, i <= M, i++,

      g[[K1 (i - 1) + 1 ;; K1 i]] =

     NIntegrate[(rm[[i]] \[CapitalOmega] /. ms[[i]]), {\[CurlyPhi]t, 0,

      2 \[Pi]}];

      F[[K1 (i - 1) + 1 ;; K1 i, K1 (i - 1) + 1 ;; K1 i]] = F0;

      ];

      W1 = Table[0, {M (M - 1)}, {M K1}];

      k = 0;

      For[i = 1, i <= M, i++,

      For[j = 1, j <= M, j++,

      If[i == j, Continue[[]];

      k = k + 1;

      {\[

     CurlyPhi]ij, \[CurlyPhi]ji, norm} =

     GetCrosSectionPhase[m[[i]], m[[j]]] // N;

      W1[[k,

      K1 (i - 1) + 4 ;;

      K1 (i - 1) + 4 +

      2 K]] = \[CapitalPhi] /. \[CurlyPhi]t -> \[CurlyPhi]ij;

      W1[[k,

      K1 (j - 1) + 4 ;;

      K1 (j - 1) + 4 +

      2 K]] = -\[CapitalPhi] /. \[CurlyPhi]t -> \[CurlyPhi]ji;

      ]

      ];

      W2 = Table[0, {4}, {M K1}];

      For[i = 1, i <= M, i++,

      W2[[1 ;; 4, K1 (i - 1) + 4 ;; K1 i]] =

     NIntegrate[

      Outer[Times, (\[CapitalPsi] /. ms[[i]]), \[

     CapitalPhi] \[Eta]t[[

      i]]], {\[CurlyPhi]t, 0, 2 \[Pi]}]

      ];

      \[Rho] = 1000;

      H = Table[0, {M K1 + 4}, {M K1 + 4}];

      H[[1 ;; M K1, 1 ;; M K1]] = F + \[Rho] Transpose[W1].W1;

      H[[M K1 + 1 ;; M K1 + 4, 1 ;; M K1]] = W2;

      H[[1 ;; M K1, M K1 + 1 ;; M K1 + 4]] = Transpose[W2];

      pfilter = -

     LinearSolve[H, g~Join~Table[0, {4}]];

      \[CapitalDelta]rf =

      Table[\[CapitalPhi].pfilter[[K1 (i - 1) + 4 ;; K1 i ]], {i, 1, M}];

      \[CapitalDelta]rf ]
